# Further insight into the bioactivity of the freshwater sponge *Ochridaspongia rotunda*

**DOI:** 10.1080/13880209.2017.1297468

**Published:** 2017-03-09

**Authors:** Aleksandra Talevska, Boris Pejin, Tanja Beric, Slavisa Stankovic

**Affiliations:** a University of St. Clement of Ohrid, Hydrobiological Institute, Ohrid, Republic of Macedonia;; b Faculty of Biology, University of Belgrade, Belgrade, Serbia;; c Department of Life Sciences, Institute for Multidisciplinary Research – IMSI, University of Belgrade, Belgrade, Serbia

**Keywords:** Bioprospection, Malawispongiidae, AChE, Alzheimer's disease

## Abstract

**Context:** Bioprospection has become a dynamic scientific field that explores novel possibilities for the implementation of natural products in medicine and pharmacy. Compared to marine species from all kingdoms, freshwater species have been highly neglected.

**Objective:** This work focuses on the screening of acetylcholinesterase inhibitory (AChE) and mutagenic activities of the acetone extract (obtained by maceration) of the freshwater sponge *Ochridaspongia rotunda* Arndt (Malawispongiidae) *in vitro*.

**Materials and methods:** AChE inhibitory activity was evaluated both in liquid (five different concentrations of the extract, from 1 to 100 μg/mL) and in solid (seven different concentrations of the extract, from 0.5 to 10.0 μg) by methods well described in literature, while mutagenicity was estimated using the Ames test (four different concentrations of the extract, from 0.106 to 1.328 mg/plate).

**Results:***Ochridaspongia rotunda* acetone extract exhibited promising AChE inhibitory activity in a dose-dependent manner both in liquid (IC_50_ 23.07 μg/mL) and in solid (1.50 μg). Furthermore, the Ames test revealed no sign of mutagenicity at any concentration tested. Its FTIR spectrum coupled with the positive Liebermann?Burchard, Salkowski and Zak color reactions (tests) indicated the presence of sterol compounds.

**Discussion and conclusion:** The screened extract may inspire a search for novel anticholinesterase therapeutic agent(s) potentially used in the treatment of Alzheimer's disease. Further research will be directed toward its detailed chemical analysis along with addressing the issue of a real producer of the natural product(s) responsible for the AChE activity observed.

## Introduction

Bioprospection has become a dynamic scientific field that explores novel possibilities for the implementation of natural products. During the last 60 years or more, much of the efforts in chemical science have been focused on metabolites from the sea (Mehbub et al. 2016; Wang et al. 2015; Zhang et al. 2017). Variety of species and environmental conditions significantly different than those in terrestrial ecosystems provided evolutionary pressure for the development of unique molecules that can be used for various pharmaceutical purposes, including the treatment of tumors and Alzheimer's disease (AD) (Blunt et al. 2016; Faulkner 1996; Pejin et al. 2013a). Among the species within the marine animal kingdom, sponges are the organisms that have attracted the most attention so far (Dionisi et al. 2012; Haefner 2003; Simmons et al. 2005).

Alzheimer's disease, which is the most common age-related neurodegenerative disorder, is manifested by a progressive loss of memory and cognition. The disease is accompanied by a deficiency in cholinergic neurotransmission. Acetylcholinesterase (AChE) is the enzyme involved in the metabolic hydrolysis of acetylcholine at cholinergic synapses in the CNS and PNS (Scott & Goa 2000).

The number of chemical compounds that have been isolated and characterized from any freshwater animal or plant species is relatively small in comparison to marine natural products. The main reason for the large distinction is that less than 3% of all the water on Earth is fresh. Most of the water that makes up the 3% is either in groundwater or locked up in ice caps and glaciers. Less than 0.01% of all water on Earth is found in freshwater rivers and lakes and this has a significant effect on the amount of biodiversity that is found in fresh water compared to marine environments. This lack of biodiversity is reflected in the number of freshwater species compared to their marine counterparts (Andersen 2012).

Although the vast majority of an estimated 15,000 sponges populate marine environments, about 150 species live in freshwater habitats such as lakes and rivers (Hooper & Van Soest 2002). In comparison to their marine counterparts, the diversity of secondary metabolites is low with lipid molecules being notable exceptions. As a matter of fact, more than 100 novel unusual and rare fatty acids, phosphonolipids and sterols have been isolated from freshwater sponges. Sterols from sponges have received attention because they present patterns of branches that distinguish them from all other living organisms (de Barros et al. 2013, 2015; Dembitsky et al. 2003; Manconi et al. 1988). Not so long ago Hu et al. (2009) isolated a new spongilipid tetracosan-1-ol-1-*O*-β-d-glucopyranoside (together with 10 known compounds including cholesterol and its derivatives) from the cosmopolitan freshwater sponge *Spongilla lacustris* Linn. (Spongillidae).

The sponge *Ochridaspongia rotunda* Arndt (Malawispongiidae) is endemic to Lake Ohrid, the oldest lake in Europe. The rounded sponge *O. rotunda* with no close relatives in the current living world inhabits the deeper zones (30–70 m depth) under conditions of constant low temperatures (Albrecht & Wilke 2008). The species was very abundant in Lake Ohrid in the 1950 s when many specimens were collected by professional fishers using nets (Hooper & Van Soest 2002).

During the screening of the biological activity of the *O. rotunda* sponge (Pejin et al. 2014) its acetone extract was investigated. Herein, we report *in vitro* AChE inhibitory and nonmutagenic activities of the aforementioned extract determined for the first time.

## Materials and methods

### Biological material

The sample of *O. rotunda* (verified by Research Professor Dr. Trajce Talevski, Hydrobiological Institute, Ohrid) was collected in Lake Ohrid in September 2014. Voucher specimen (LO ORO 006) has been deposited in the Hydrobiological Institute, Ohrid, Republic of Macedonia.

### Extraction

The air dried parts of *O. rotunda* (drying outside, in the shade; ca 2 weeks) were ground (4 g; without using homogeniser) and extracted thrice with acetone (300 mL; solid-liquid extraction – maceration) for 1 h at room temperature (yield, 7%). Afterward the extract was evaporated to dryness and stored at -20 °C until further use.

### Color reactions

The Liebermann–Burchard, Salkowski and Zak reactions (tests) were done as previously described (Goad & Akihisa 1997; Xiong et al. 2007).

### FTIR analysis

Fourier-transform infrared (FTIR) spectrum of *O. rotunda* acetone extract was recorded in the attenuated total reflection mode (ATR) using a Nicolet 6700 FTIR Spectrometer (Thermo Scientific).

### The Ames test

The Ames test was done as previously described (Maron & Ames 1983). Three *Salmonella typhimurium* histidine-auxotrophic strains (namely, TA98, TA100 and TA102) were used. Cultures were prepared from master plates, grown in LB liquid medium and used after overnight incubation at 37°C (late exponential growth phase). Each experiment included negative control (distilled water), solvent control (dimethyl sulfoxide), appropriate positive control and four concentrations (0.106-1.328 mg/plate) of the extract tested. The extract was assessed in three independent experiments, in absence and presence of the S9 metabolic activation system from rat livers, using duplicate plates for the sample. In short, 0.25 mL of the extract, 0.1 mL of bacterial culture and 0.3 mL of S9 mix were added to 3 mL of molten top agar supplemented with histidine/biotine, at 42 °C and poured onto Vogel Bonner E minimal glucose agar plates. The plates were incubated for three days at 37 °C. Revertant colonies were counted, and the background lawn was inspected for signs of toxicity. 4-Nitroquinoline-*N*-oxide (1 μg/plate)/without the S9 fraction/and ethidium bromide (50 μg/plate)/in presence of the same fraction/were selected as positive controls, respectively.

### Acetylcholinesterase inhibitory activity test

#### In liquid

*In vitro* determination of AChE inhibitory activity was done by the method of Ellman et al. (1961) which is adapted for the use in 96-well microtiter plate. Acetylcholine iodide originating from electric eel is used as an artificial substrate for the AChE enzyme which degrades this compound to acetate and tioholin. Subsequently, dithiobenzoate reacts with tioholin producing yellow color measured spectrophotometrically. The mixture was incubated with five different concentrations of the extract (extract 1–100 μg/mL) for 10 min. Galanthamine was used as a standard, while the mixture without the extract tested was used as a control (100% enzyme activity). The experiment was carried out in triplicate. The result is expressed as an IC_50_ value, i.e. in the form of the concentration at which the 50% of enzyme inhibition was observed.

#### In solid

The assay was performed as previously described (Marston et al. 2002). It is a simple and rapid bioautographic enzyme assay. The test relies on the cleavage of 1-naphthyl acetate by AChE to form 1-naphthol, which in turn reacts with Fast Blue B salt to give a purple-colored diazonium dye. AChE inhibitory activity was detected by the appearance of a white spot on a purple background. Galanthamine was used as positive control in this test as well. This experiment was also carried out in triplicate. Briefly, a stock solution of AChE (1000 U in 150 mL of Trishydrochloric acid buffer pH 7.8) was obtained, which was stabilized adding bovine serum albumin (150 mg). A 10 μL aliquot of each solution of the extract (seven different concentrations, 0.5–10.0 μg) was applied to the TLC plate, dried to remove the solvent, and then sprayed with enzyme stock solution. For incubation of the enzyme, the plate was kept at 37 °C for 20 min in a humid atmosphere. For the detection of the enzyme, solutions of 1-naphthyl acetate (250 mg in 100 mL of ethanol) and of Fast Blue B salt (400 mg in 160 mL of distilled water) were mixed and sprayed onto the plate. Acetylcolinesterase inhibitory activity was detected by a white spot on a purple background after 1–2 min. Galanthamine was used as a standard in this test as well. This experiment was also carried out in triplicate. The result is expressed as the minimum amount of the extract at which the enzyme was inhibited.

## Results

*Ochridaspongia rotunda* acetone extract exhibited promising AChE inhibitory activity both in liquid (IC_50_ 23.07 μg/mL) and in solid (1.50 μg) conditions. Furthermore, the Ames test showed no sign of mutagenicity (i.e. no increase in the number of revertants) in any of the treated strains up to the highest tested concentration, either in the presence or absence of S9 (Table 1). In addition to this, the toxicity of the extract, estimated as a decrease in the background lawn and revertant frequency, was not observed at any tested concentration. However, statistically significant increases (number of revertant colonies; *p* < 0.05), noted for the positive controls, validated both the sensitivity of the test system and activity of the S9 mix (three *Salmonella typhimurium* histidine-auxotrophic strains TA98, TA100, and TA102).

**Table 1.** The Ames test: *Ochridaspongia rotunda* acetone extract.

	Revertant colonies (CFU/plate)^a^					
	TA98		TA100		TA102	
Extract	− S9	+ S9	− S9	+ S9	− S9	+ S9
Control	16 ± 4	20 ± 2	113 ± 11	105 ± 13	250 ± 21	276 ± 26
DMSO (50 μL/plate)	19 ± 5	18 ± 3	103 ± 5	120 ± 16	298 ± 19	286 ± 14
0.106 mg/plate	16 ± 2	33 ± 6	119 ± 6	102 ± 9	340 ± 18	355 ± 5
0.265 mg/plate	22 ± 3	28 ± 4	165 ± 13	133 ± 11	306 ± 10	290 ± 9
0.531 mg/plate	20 ± 2	24 ± 5	103 ± 14	90 ± 3	308 ± 15	261 ± 20
1.328 mg/plate	24 ± 6	27 ± 2	108 ± 2	140 ± 10	281 ± 7	296 ± 16
4-NQO (1 μg/plate)	–	–	856 ± 87*	811 ± 48*	1917 ± 195*	2159 ± 67*
EtBr (50 μg/plate)	23 ± 6	648 ± 68*	–	–	–	–

a Mean values ± SD of three experiments.

* Significantly different (*p* < 0.05).

Finally, Fourier transform infrared (FTIR) spectrum of *O. rotunda* acetone extract suggested the possible presence of sterol compounds (hydroxyl and methylene groups 3462.2 cm^−1^ and 2854.7 and 2927.7 cm^−1^, respectively) that was confirmed by the positive Liebermann–Burchard, Salkowski and Zac colored reactions (tests).

## Discussion

Rivastigmine (a synthetic analogue of the alkaloid physostigmine), an acetylcholinesterase inhibitor, has been the first approved 'smart drug' in the EU zone. However, this drug has numerous side effects including abdominal pain, headache, nausea, and vomiting, weight loss, and anorexia which may be accompanied by depression, fatigue, and tremor. Donepezil and galanthamine, the other two alkaloid drugs frequently used in the treatment of AD, are also not without side effects. Furthermore, huperzine A, a naturally occurring sesquiterpene alkaloid used as AD drug, may cause mild to moderate cholinergic side effects. There is actually a profound interest in the worldwide research community for new AChE inhibitors not belonging to this structural class (Graham 2005).

Our recent findings in the field have shed some light on the avarol scaffold (de Rosa & Tommonaro 2012; Tommonaro et al. 2015). Avarol (sesquiterpene hydroquinone with a rearranged drimane skeleton) was firstly isolated from the Mediterranean sponge *Dysidea avara* Schmidt (Dysideidae) along with minor amounts of its oxidized derivative avarone (Minale et al. 1974; De Rosa et al. 1976). The AChE inhibitory test indicated a moderate inhibitory activity (1 μg, in solid) for all thio-avarol derivatives with a carboxylic group in the molecule, namely, avarol-3″-thiosalicylate, avarol-3″-thiolactate, avarol-4″-thiolactate, avarol-3″-thioglycolate, avarol-4″-thioglycolate and avarol-3″-thiobenzoate (Pejin et al. 2008). Further, both avarol-3″-thiosalicylate and thiosalycil-prenyl-hydroquinones were found to be non-hepatotoxic anticholinesterasic agents exhibiting a good neuroprotective effect on the decreased viability of SHSY5Y human neuroblastoma cells induced by oligomycin A/rotenone and okadaic acid (Tommonaro et al. 2016). Additionally, the phytochemical investigation conducted on a foliose lichen specimen, *Lobaria pulmonaria* L. Hoffm. (Lobariaceae), led to the isolation of a new depsidone in the form of its diacetate derivative which showed a moderate acetylcholinesterase inhibitory activity (1 μg) *in vitro*. This is actually the first record of identified depsidone structure in the search for these inhibitors (Pejin et al. 2013b; Ece & Pejin 2015).

According to the best of our knowledge, there are no previous records of AChE inhibitory bioactivity of the freshwater sponges inhabiting the Western Balkans region. On the other hand, a recent report describing the anticholinesterase activity of 45 Mauritian marine sponges showed that these sponges are rich sources of extracts with significant AChE inhibitory activity. The species with the strongest activities were found to be *Pericharax heteroraphis* Poléjaeff (Leucettidae) and *Amphimedon navalis* Pulitzer-Finali (Niphatidae) reaching inhibitory activities of 90 ± 5 and 96 ± 1%, respectively (ethyl acetate extracts), at the concentration of 0.1 mg/mL (Beedessee et al. 2013).

Sterol compounds have also been reported among non-alkaloid based AChE inhibitors. For example, sitosterol-3-*O*-β-d-glucoside and 6″-palmitoyl-sitosterol-3-*O*-β-d-glucoside showed a profound AChE inhibitory activity *in vitro* (65.0 ± 1.3 and 72.8 ± 1.5%, respectively) (Pereira et al. 2016). Phlorofucofuroeckol-A, a sterol compound from the seaweed *Ecklonia stolonifera* Okamura (Lessoniaceae) exhibited inhibitory potential against AChE with an IC_50_ value of 4.89 ± 2.28 μM (Yoon et al. 2008). In addition to this, some Δ^5^-3β,7β-dihydroxy and Δ^5^-3β,7α-dihydroxy sterols have been found to possess inhibitory potential in a concentration-dependent manner against butyrylcholinesterase (Lu et al. 2006).

Bacteria are believed to play an important role in the fitness and biochemistry of sponges. For example, the freshwater sponge *Ephydatia fluviatilis* Linn. (Spongillidae) hosts both transient and persistent *Pseudomonas* symbionts displaying antimicrobial activities of potential biotechnological value (Gaikwad et al. 2016; Keller-Costa et al. 2014). Therefore, the origin of *O. rotunda* bioactive compounds should be carefully addressed. The sponge symbiotic microorganism(s) as its/their real producer(s) may actually facilitate the way toward new lead compounds and/or drugs to a great extent. Not to mention the additional value of such a finding from the ecological point of view.

Taken all together, further research will be directed toward a detailed chemical analysis of this sponge species (including both isolation and identification of *O. rotunda* natural product(s) responsible for the bioactivity observed) along with determination of their real producer(s) – the sponge itself and/or its symbiotic microorganisms with focus on bacteria.
